# Older patients with primary central nervous system lymphoma: Survival and prognostication across 20 U.S. cancer centers

**DOI:** 10.1002/ajh.26919

**Published:** 2023-04-05

**Authors:** Kevin A. David, Suchitra Sundaram, Seo-Hyun Kim, Ryan Vaca, Yong Lin, Samuel Singer, Mary-Kate Malecek, Jordan Carter, Adam Zayac, Myung Sun Kim, Nishitha Reddy, Douglas Ney, Alma Habib, Christopher Strouse, Jerome Graber, Veronika Bachanova, Sidra Salman, Jean Alyxa Vendiola, Nasheed Hossain, Mazie Tsang, Ajay Major, David A. Bond, Prashasti Agrawal, Angel Mier-Hicks, Pallawi Torka, Priya Rajakumar, Parameswaran Venugopal, Stephanie Berg, Michael Glantz, Samuel A. Goldlust, Matthew Folstad, Pallavi Kumar, Thomas A. Ollila, Johnny Cai, Stephen Spurgeon, Alex Sieg, Joseph Cleveland, Julie Chang, Narendranath Epperla, Reem Karmali, Seema Naik, Peter Martin, Sonali M. Smith, James Rubenstein, Brad Kahl, Andrew M. Evens

**Affiliations:** 1 Rutgers Cancer Institute of New Jersey, New Brunswick, New Jersey, USA; 2 Roswell Park Cancer Institute, Buffalo, New York, USA; 3 Rush University Medical Center, Chicago, Illinois, USA; 4 Penn State Cancer Institute, Hershey, Pennsylvania, USA; 5 John Theurer Cancer Center, Hackensack, New Jersey, USA; 6 Washington University in St. Louis, St. Louis, Missouri, USA; 7 Brown University, Providence, Rhode Island, USA; 8 Oregon Health & Science University, Portland, Oregon, USA; 9 Vanderbilt University, Nashville, Tennessee, USA; 10 University of Colorado, Aurora, Colorado, USA; 11 University of Minnesota, Minneapolis, Minnesota, USA; 12 University of Iowa, Iowa City, Iowa, USA; 13 University of Washington, Seattle, Washington, USA; 14 Loyola University Medical Center, Maywood, Illinois, USA; 15 University of California, San Francisco, California, USA; 16 University of Chicago, Chicago, Illinois, USA; 17 Division of Hematology, Ohio State University, Columbus, Ohio, USA; 18 Weill Cornell Medical College, New York City, New York, USA; 19 University of Wisconsin, Madison, Wisconsin, USA; 20 Northwestern University, Chicago, Illinois, USA

## Abstract

There is a paucity of large-scale data delineating outcomes and prognostication of older patients with primary central nervous system lymphoma (PCNSL). We retrospectively analyzed 539 newly-diagnosed PCNSL patients ages ≥60 years across 20 U.S. academic centers. The median age was 70 years (range 60–88); at least one geriatric syndrome was present in 46%; the median Cumulative Index Ratings Scale-Geriatrics (CIRS-G) score was 6 (range, 0–27); and 36% had impairment in activities of daily living (ADL). The most common induction regimens were high-dose methotrexate (HD-MTX) ± rituximab; methotrexate, temozolomide, rituximab (MTR); and rituximab, methotrexate, procarbazine, vincristine (R-MPV). Overall, 70% of patients achieved remission, with 14% undergoing consolidative autologous stem cell transplant (ASCT) and 24% receiving maintenance. With 58-month median follow-up, median progression-free survival (PFS) and overall survival (OS) were 17 months (95% CI 13–22 months) and 43 months (95% CI 31–56 months), respectively. Three-year PFS and OS were highest with MTR (55% and 74%, respectively). With single-agent methotrexate ± rituximab, 3-year PFS and OS were 30% (*p*= .0002) and 47% (*p* = .0072). On multivariate analysis, increasing age at diagnosis and Cooperative Oncology Group (ECOG) performance status (PS) was associated with inferior PFS; age, hypoalbuminemia, higher CIRS-G score, and ECOG PS adversely affected OS. Among patients receiving maintenance, 3-year PFS was 65% versus 45% without maintenance (*p*= 0.02), with 3-year OS of 84% versus 61%, respectively (*p*= .0003). Altogether, outcomes in older PCNSL patients appeared optimized with HD-MTX combination induction regimens and maintenance therapy. Furthermore, several prognostic factors, including geriatric measures, were associated with inferior outcomes.

## 1 | INTRODUCTION

Primary central nervous system lymphoma (PCNSL) is a highly aggressive non-Hodgkin lymphoma confined to the brain, spine, eyes, and/or leptomeninges. The risk of PCNSL increases exponentially with age, and the disease incidence has been increasing overall, largely by the rising incidence in older patients.^1^ However, the long-term survival of patients older than ages 60 years has not appeared to improve, remaining relatively modest over the past few decades, especially for patients over 70 years.^1,2^ The cornerstone of treatment for PCNSL has included induction therapy with high-dose methotrexate (HD-MTX) at a dose of 3 to 8 g per square meter.^3^ However, the tolerability of HD-MTX can be challenging for older patients, especially related to renal toxicity. Most randomized data regarding treatment options in PCNSL stems from studies in younger patients. Thus, the optimal treatment strategy in older PCNSL patients remains largely unclear.

Investigators have examined a number of induction regimens in PCNSL building upon an HD-MTX backbone, including combinations incorporating procarbazine, vincristine, and rituximab (R-MPV),^4^ as well as combinations with temozolomide.^5^ The randomized phase II International Extranodal Lymphoma Study Group (IELSG) 20 study showed that adding high-dose cytarabine to methotrexate was associated with improved progression-free survival (PFS) compared with methotrexate monotherapy.^6^ The same group later conducted the IELSG32 study of the MATRix regimen, which added thiotepa and rituximab to a backbone of HD-MTX and cytarabine and compared this combination to methotrexate and cytarabine, with or without rituximab.^7^ However, there were few patients over the age of 60 years, and this study did not include patients older than 70 years.

Controversy also exists regarding the best consolidative strategy following induction therapy. High-dose chemotherapy with autologous stem cell transplantation (ASCT) has been studied mainly in patients younger than 60 years, and concerns about tolerability in older patients are significant.^8^ Whole brain radiotherapy is another potential consolidative strategy, but significant concerns about adverse neurocognitive events in older patients temper enthusiasm about this approach.^3^ In addition, most analyses of older PCNSL patients have been relatively small, and clinical prognostication factors are poorly studied. This includes the potential impact of geriatric assessments, with strong evidence of benefit in older cancer patients.^9,10^

Herein, we report a multicenter collaboration that investigated a large retrospective cohort of 539 adult patients ages ≥60 years with newly diagnosed PCNSL managed during a recent 11-year period across 20 U.S. academic cancer centers. We investigated detailed clinical and disease-related characteristics, including geriatric assessments, and also analyzed treatment patterns. We also evaluated the relationship between these factors and patient outcomes, with attention to key clinical prognostic factors associated with survival.

## 2 | METHODS

We conducted a multi-center retrospective study of older adult patients (age ≥ 60 years) with untreated PCNSL diagnosed between January 2008 and January 2019 at 20 U.S. academic cancer centers. Both immunocompetent and immunocompromised patients were included. The study was approved by the institutional review boards of all sites. Diagnosis was established by local institutional hematopathology expert review; central pathologic review was not done. Staging and therapeutic decisions were made in accordance with local institutional standards.

We collected detailed demographic, clinicopathologic, and outcomes data using a standardized protocol. Performance status (PS) was assigned according to the Eastern Cooperative Oncology Group (ECOG) scale. Serum lactate dehydrogenase (LDH) was standardized relative to each institution's upper limit of normal (ULN). A number of geriatric assessment tools are currently in use; for this study, we collected information on three of these: CIRS-G,^11^ presence of any geriatric syndrome (defined as dementia, delirium, depression, osteoporosis, incontinence, falls, failure to thrive, or neglect/abuse), and impairment in activities of daily living (ADL), defined as bathing, dressing, toileting, transferring, feeding, and continence.

PFS was defined according to the 2005 International Working Group criteria as time from diagnosis until disease progression, recurrence, or death from any cause.^12^ Overall survival (OS) was calculated from diagnosis until death or last follow-up. Treatment-related mortality (TRM) was defined as death from any cause other than lymphoma because of a treatment-related adverse event (causation was determined by local investigators retrospectively). PFS and OS rates were estimated by Kaplan–Meier with differences assessed by log-rank test, with hazard ratios reported for comparisons between patient groups.

For descriptive purposes, the association between all pre-treatment patient characteristic variables and disease-related variables was examined by univariate modeling. Clinicopathologic characteristics were compared between patient groups using rank-sum or Fisher's exact tests. Median follow-up was determined by reverse Kaplan–Meier analysis. To determine factors to be included in a PCNSL-specific and clinically useful prognostic model, we then conducted a multivariate analysis. Graphical inspection of survival curves was used to facilitate the assessment of the association between clinical factors and PFS/OS. Additionally, Harrell's C concordance coefficients were used to select optimal cutoff values for age and CIRS-G score.

## 3 | RESULTS

Baseline disease and clinical characteristics are presented in Table 1 with associated univariate associations for PFS and OS. Median age at diagnosis was 70 years (range 60–88), with 46% of patients ages 60–69, 43% ages 70–79, and 11% ages 80 or older. Forty-one percent of patients had an ECOG PS of 0–1; 45% had ECOG PS of 2–4, and ECOG PS was unknown in 13%. Creatinine clearance at diagnosis was available in 60% of patients. In this group, median creatinine clearance was 81 ml/min (range 16–227). Baseline hemoglobin was available in 95% of patients, with 8% of all subjects noted to be anemic with hemoglobin <10 g/dl. Hypoalbuminemia (albumin less than 3.5 g/dl) was noted in 40% of the study population; 8% of subjects had missing data regarding albumin at diagnosis.

Cerebrospinal fluid was involved in 13% of patients (27% unknown/untested), and 10% exhibited ocular involvement (19% unknown/untested). Fifty percent of patients had more than one site of parenchymal brain involvement. Cerebral involvement predominated in 66% of patients, cerebellar in approximately 4%, while deep structure (thalamus, basal ganglia, etc) in 16%. Histology was diffuse large B cell lymphoma (DLBCL) in 95% of cases. The remaining cases included a mixture of other histologies, including high-grade B cell lymphoma, unspecified low-grade lymphomas, intravascular B cell lymphoma, blastoid mantle cell lymphoma, and T cell lymphoma. The cell of origin was the germinal center (GC) in 13% of cases, non-GC in 41%, and unknown in 46%. At least one geriatric syndrome was present in 46% of patients; 36% had impairment in at least one ADL at diagnosis; and the median CIRS-G score was 6 (range 0–27).

### 3.1 | Initial therapy

Primary treatment was chemotherapy in 489 (91%) patients; while radiation therapy was used as primary treatment in 30 (5%) patients. The remaining 5% of patients died without treatment or pursued hospice care. Of the 489 patients who received chemotherapy, 413 (84%) received rituximab as part of their treatment. Among these 489, the most common induction regimens were single-agent HD-MTX either with or without rituximab in 35% of patients, MPV (methotrexate, procarbazine, vincristine) in 32% of patients, and MTR (methotrexate, temozolomide, rituximab) in 21% of patients. Other regimens included methotrexate/cytarabine in 3%, single-agent rituximab in 3%, temozolomide in 2%, MATRIX (methotrexate, cytarabine, thiotepa, and rituximab) in 2%; and bendamustine/rituximab, R-CHOP (rituximab, cyclophosphamide, doxorubicin, vincristine, prednisone), HyperCVAD/MA (cyclophosphamide, vincristine, doxorubicin, dexamethasone/methotrexate, cytarabine), and intravitreal chemotherapy were each used in less than 1% of patients. Among patients with ocular involvement, ocular-targeted therapies were used infrequently. Specifically, among 13 patients with ocular involvement alone, one patient received intra-ocular methotrexate in combination with systemic treatment and four patients received bilateral orbital radiation as primary therapy. Among 38 patients with both ocular and brain parenchymal involvement, three received intra-ocular methotrexate combined with systemic therapy and one received intra-ocular methotrexate alone.

Response to initial therapy was complete response (CR) in 54%, stable disease in 5%, partial response (PR) in 16%, progression in 15%, and unknown in 10%. Per regimen, the median number of cycles administered and range were as follows: MATRIX median 4 cycles (range 2–6), (R)MPV median 5 cycles (range 1–9), single-agent methotrexate median 4 cycles (range 1–15), and MTR median 5 cycles (range 1–12). Among patients who received methotrexate as part of induction treatment, median methotrexate dose was 3500 mg/m^2^ (range 500–8000 mg/m^2^). Mean initial methotrexate dose was 3174 mg/m^2^ in patients receiving MPV; 5229 mg/m^2^ in patients receiving MR, and 5054 mg/m^2^ in patients receiving MTR. The difference in methotrexate dose between the MPV and MR groups, as well as between the MPV and MTR groups was significant (*p* < .0001), but not significantly different between the MR and MTR groups (*p* = .4684). In the group of patients treated with (R)MPV, the median cumulative methotrexate dose was 14 000 mg/m^2^; single-agent methotrexate 20 000 mg/m^2^ and MTR 18000 mg/m^2^. Treatment was stopped due to toxicity in 112 patients (21%). Among these patients, nephrotoxicity prompted treatment cessation in 28%, infection in 20%, neurotoxicity in 5%, and hepatotoxicity in 4%. In the remaining cases of discontinuation, a variety of other causes contributed or the cause was not documented. Within this group, 56% received no further therapy and died either of therapy complications or progressive lymphoma, 26% went on to get other chemotherapy, 13% received radiation, and 5% remained alive without further therapy. TRM was 6.9%. Among those patients who died from treatment-related causes, 46% died from infection, 8% from organ failure, 5% from leukoencephalopathy, and 3% from intracranial hemorrhage, with the cause undocumented/unknown in 38%.

### 3.2 | Post-induction therapy

We analyzed characteristics and post-induction treatment patterns among the 376 patients (70% of the entire cohort) who achieved either CR or PR to initial therapy. The median age among these patients was 69 years (range 60–88). Among this subgroup, 87 patients (23%) were in PR and 289 (77%) were in CR. Fifty-one patients (14%) underwent autologous stem cell transplant, with median age of 66 years (60–77) and ECOG PS 0–1 in 73% of ASCT patients. At the time of ASCT, 40 (78%) were in CR and 11 (22%) were in PR. The majority of patients received thiotepa-containing conditioning. Conditioning regimens were carmustine/thiotepa (57%), thiotepa/busulfan/cyclophosphamide (14%), busulfan/cyclophosphamide (8%), busulfan/cyclophosphamide/etoposide (2%), busulfan/thiotepa (2%), cyclophosphamide/total body irradiation (2%), thiotepa/etoposide/cytarabine/melphalan (2%) and unknown (13%). Three patients (6%) died due to transplant-related complications.

Twenty patients with a median age 68 years (range, 62–75) received consolidation radiation therapy. Thirty-eight patients received consolidation cytarabine without subsequent ASCT. Post-induction maintenance was given to 90 patients (24%) with a median age of 72 years (60–86). The most common maintenance treatments were temozolomide (31%), lenalidomide (23%), and methotrexate (17%). Of note, of the 29 patients who received temozolomide maintenance, 12 (41%) had received prior MTR induction, and 11 (38%) received MPV induction. At the start of maintenance, 77 patients (83%) were in CR, and 15 (17%) were in PR. Only two patients received maintenance therapy following ASCT.

### 3.3 | Treatment for relapsed/refractory disease

A total of 238 patients had a relapse or primary refractory disease. Among these patients, a variety of treatments were employed. The most common regimens used were radiation therapy (49 patients), single-agent methotrexate (45 patients), single-agent temozolomide (21 patients), MTR (13 patients), and single-agent cytarabine (seven patients). A small number of patients received newer agents, such as ibrutinib and lenalidomide, given to six patients each. Among patients receiving radiation for relapsed/refractory lymphoma, the CR rate was 33% and PR rate was 14%. Among patients treated with single-agent methotrexate, the CR rate was 40% and PR rate was 13%. With temozolomide alone, the CR rate was 19%. With ibrutinib, the CR rate was 50%.

### 3.4 | Survival

**FIGURE 1:**
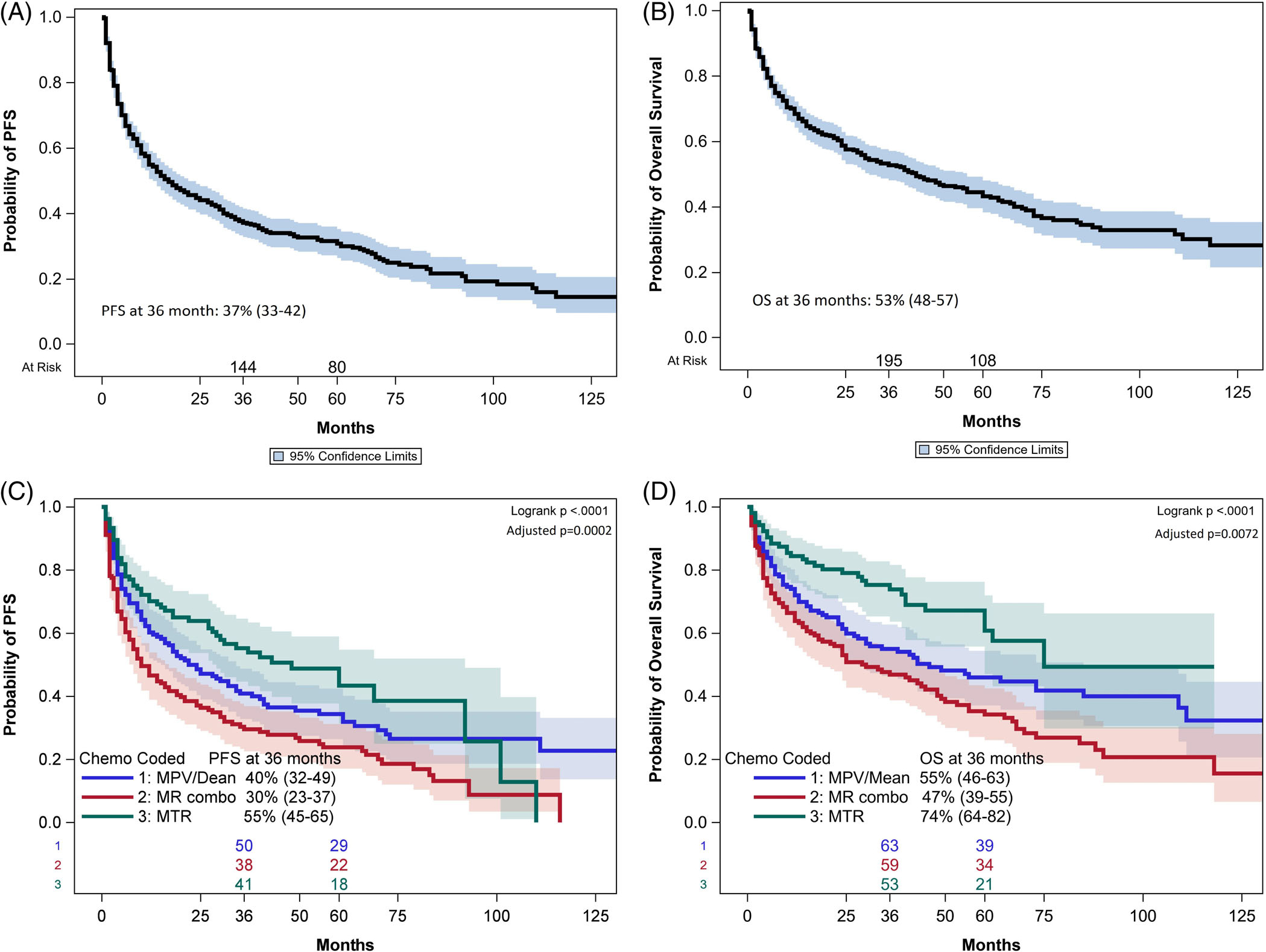
(A) Median PFS for the entire cohort of 539 patients was 17 months (95% CI 13–22 months). PFS at 36 months was 37% (955 CI 33–42). (B) Median OS was 43 months (95% CI 31–56 months). OS at 36 months was 53% (95% CI 48–57). (C). PFS for each of the three most common induction regimens, adjusted for significant pre-treatment variables. PFS was highest for patients treated with the MTR regimen. (D). OS for each of the three most common induction regimens, adjusted for significant pre-treatment variables. OS was highest for patients treated with the MTR regimen.

With median follow-up of 58 months, median PFS was 17 months (95% CI 13 to 22 months, Figure 1A), and median OS was 43 months (95% CI 31 to 56 months, Figure 1B). PFS and OS for patients treated with the three most common induction regimens (methotrexate ±rituximab, MPV, and MTR) are depicted in Figure 1C,D, respectively. Three-year PFS and OS were highest for patients treated with MTR at 55% (95% CI 45–65%) and 74% (95% CI 64–82%), respectively. For patients treated with MPV, 3-year PFS, and OS were 40% (95% CI 32–49%) and 47% (46–63%), respectively, and patients treated with methotrexate without other chemotherapy had a 3-year PFS of 30% (95% CI 23–27%) and 3-year OS of 47% (95% CI 39–55%). These differences in survival between the three treatment groups persisted when adjusted for pre-treatment patient and disease characteristics. The absence of rituximab in initial treatment, controlling for pre-treatment prognostic factors, was associated with worse PFS (HR 1.71, *p* < .0001, 95% CI 1.33–2.20), and OS (HR 1.75, *p* = .0004, 95% CI 1.28–2.38).

### 3.5 | Prognostication

On univariate analysis (Tables 1 and 2), age, creatinine clearance, hemoglobin level, albumin, CIRS-G score, presence of a geriatric syndrome, and impairment in ADLs significantly affected both PFS and OS. Elevated LDH significantly affected OS, but not PFS. On multivariate analysis incorporating pre-treatment characteristics, we identified that increasing age at diagnosis and worse ECOG PS were associated with inferior PFS (Table 3). Older age at diagnosis, hypoalbuminemia, higher CIRS-G score, and worse ECOG PS had an adverse association with OS (Table 3) Furthermore, we analyzed the impact of age by Harrel's C analysis and found that the optimal age for both PFS and OS was 71 years. On multivariate analysis of PFS, controlling for ECOG PS, HR for age greater than 71 years was 1.72 (*p* < .0001, 95% CI 1.37–2.16).

**FIGURE 2:**
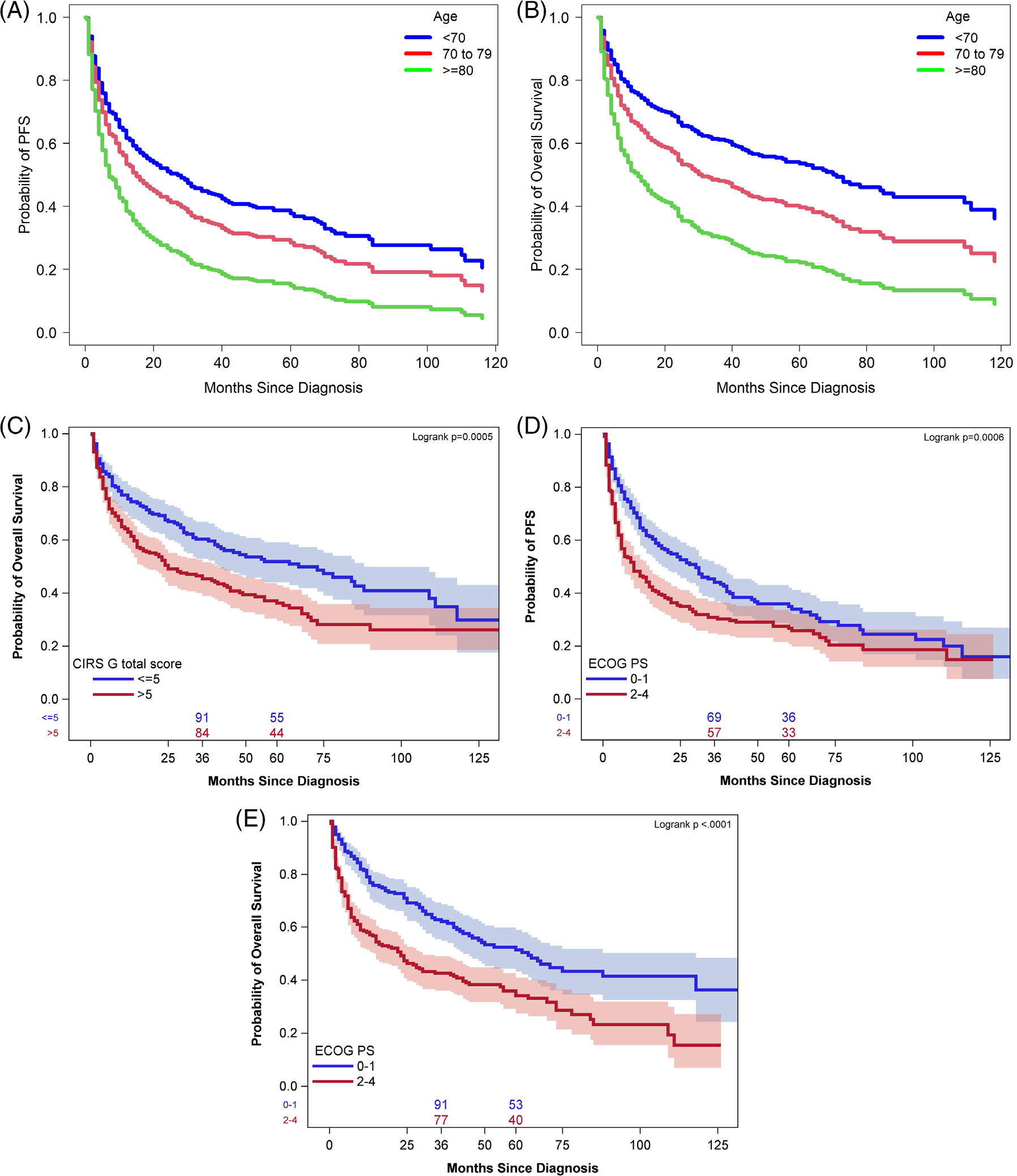
(A). PFS by age group at diagnosis. PFS was best for patients 60–70 years old at diagnosis and worse for those aged 80 and older. (B). OS by age group at diagnosis. OS was best for patients 60–70 years old at diagnosis and worse for those aged 80 and older. (C) CIRS-G score above 5 was associated with lower OS. Impaired ECOG PS was associated with worse PFS (D) and OS (E).

On multivariate analysis of OS, controlling for albumin level, CIRS-G score, and ECOG PS, the HR for age greater than 71 years was 1.94 (*p* < .0001, 95% CI 1.48–2.56). Survival delineated by age groups are shown in Figure 2A,B. Patients 80 years and older had the worst survival, while patients 60–70 years had the best. As CIRS-G score increased, OS worsened (Figure 2C). We analyzed the impact of CIRS-G score on OS by Harrel's C analysis and found that a score of less than or equal to 5 was associated with improved OS, with HR of 0.646 (*p* = .0007, 95% CI 0.503–0.831) (Figure 2C). Patients with impaired ECOG PS (2–4) had worse PFS and OS compared to those with better PS (Figures 2D,E).

**FIGURE 3:**
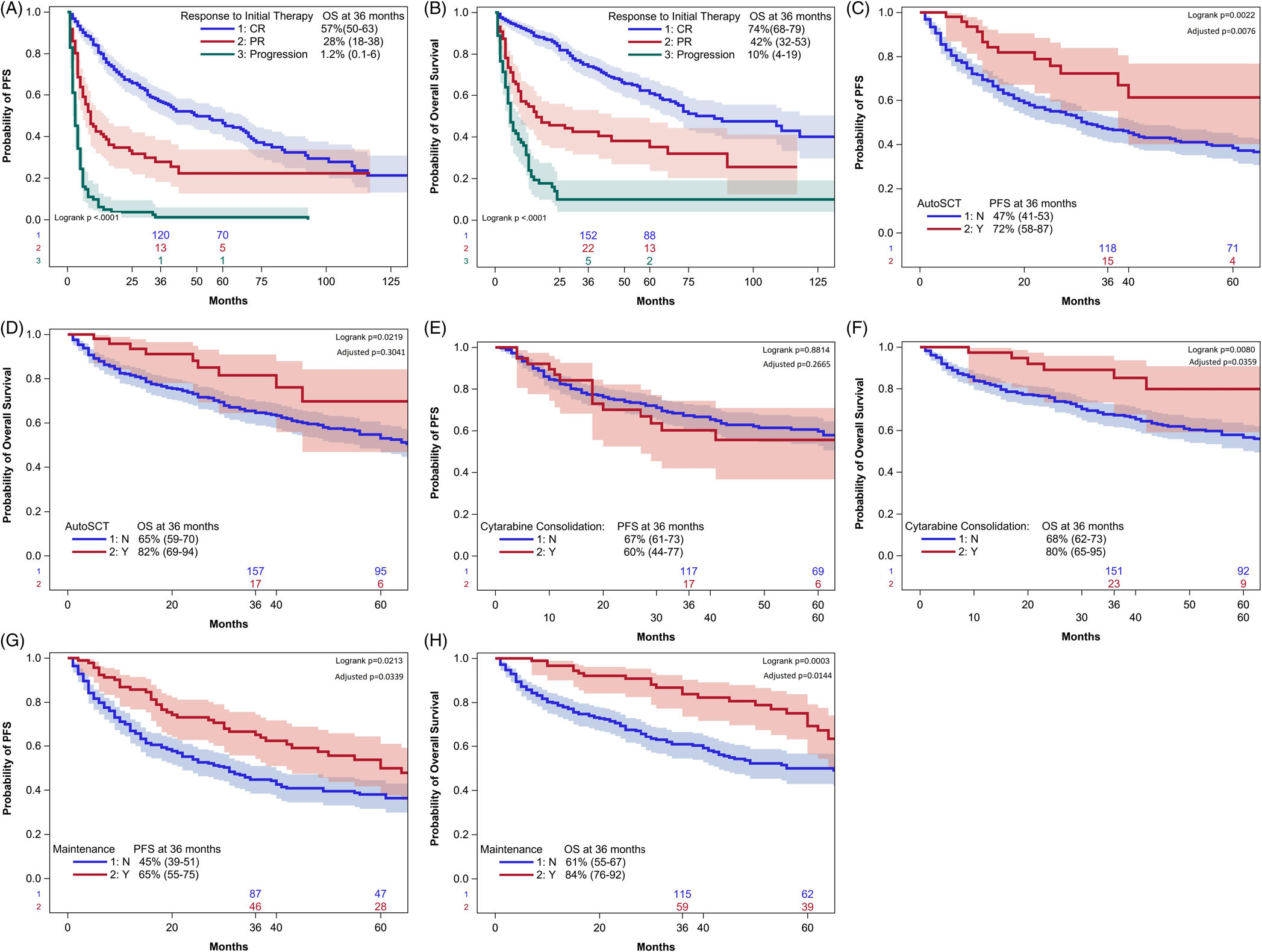
(A) Response to initial therapy significantly impacted survival. Patients who experienced CR after induction had the longest PFS, while patients with disease progression had drastically worse outcomes. (B) A similar trend was seen with regard to OS. When adjusted for pre-treatment variables, consolidative autologous SCT resulted in a statistically significant improvement in PFS (C), but not OS (D). Cytarabine consolidation did not affect PFS (E) but was associated with improved OS (F) Maintenance therapy resulted in improvements in both PFS (G) and OS (H).

Additionally, as shown in Figure 3A,B, response to initial therapy strongly impacted long-term survival. Patients who experienced CR after induction had the highest 3-year PFS (57%, 95% CI 50–63%) and OS (74%, 95% CI 68–79%). Patients with progression after initial therapy had a 3-year OS of only 10% (95% CI 4–19%). We also analyzed the impact of increasing the initial methotrexate dose in 500 mg/m^2^ intervals on survival. On multivariate analysis, increasing methotrexate improved PFS (HR 0.973, *p* = .0365, 95% CI 0.948–0.998) but not OS (HR 0.999, *p* = .6858, 95% CI 0.993–1.005). Additionally, we found that every one-gram increase in the *cumulative* methotrexate dose was associated with increased PFS (HR 0.979, 95% CI 0.968–0.989, *p* = .0001) and OS (HR 0.975, 95% CI 0.961–0.990, *p* = .0008). PFS was adjusted for age and ECOG PS, and OS was adjusted for age, albumin, CIRS-G score, and ECOG PS.

When analyzing for prognostic factors among only the 376 patients in CR or PR *after induction*, factors on multivariate analysis associated with inferior PFS were advancing age (HR 1.048, *p* = .0046, 95% CI 1.014–1.082), higher Ki-67 (HR 1.017, *p* = .0157, 95% CI 1.003–1.032) and presence of geriatric syndrome (HR 1.72, *P* = .0186, 95% CI 1.09–2.69). Advancing age (HR 1.043, *P* = .0105, 95% CI 1.010–1.078), impaired creatinine clearance (1.009, *p* = .0149, 95% CI 1.002–1.02), increased CIRS-G score (HR 1.054, *p* = .0185, 95% CI 1.009–1.100) and worse ECOG PS (e.g., PS 1 vs 3 HR 1.96, *p* = .0318, 95% CI 1.06–3.64) were associated with inferior OS.

**FIGURE 4:**
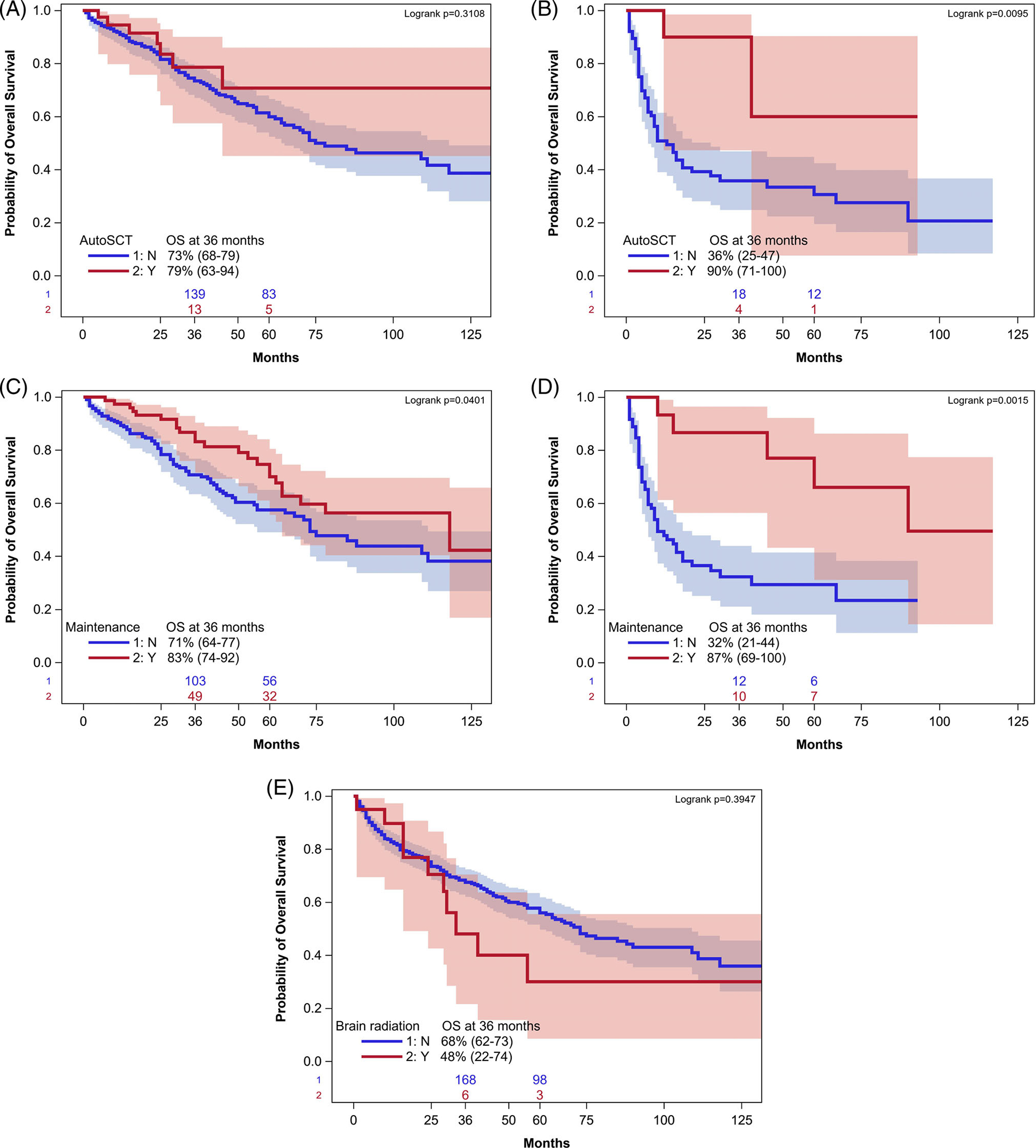
Consolidative autologous stem cell transplant and maintenance therapy appeared to have a larger benefit on OS for patients in PR at the end of induction compared to CR. OS is depicted for patients in CR (A) and PR (B) at the time of ASCT. OS is depicted for patients who received maintenance therapy in CR (C) and PR (D). Consolidative radiation therapy did not improve OS (E).

The 3-year PFS among patients undergoing ASCT was 72% versus 47% among those who did not (*p* = .002) with 3-year OS 82% versus 65%, respectively (*p* = .02); these survival improvements persisted for PFS, but not for OS when adjusted for the aforementioned prognostic factors (Figure 3C,D, respectively). Consolidation cytarabine was not associated with any improvement in PFS (3-year PFS 60% with cytarabine versus 67% without cytarabine, adjusted *p* = .2665) (Figure 3E). However, cytarabine was associated with improved OS, with the improvement persisting even when adjusted for pre-treatment variables (Figure 3F). Among patients who received maintenance therapy, the 3-year PFS was 65% versus 45% with no maintenance (*p* = .02), with 3-year OS of 84% versus 61%, respectively (*p* = .0003), with both remaining significant on adjustment for pre-treatment prognostic factors identified as prognostic on multivariate analysis (Figure 3G, H, respectively). Patients in PR appeared to benefit more from either ASCT or maintenance therapy compared with those in CR after induction, although this analysis was limited by the small number of PR pts (Figure 4A–D). Finally, consolidative radiation therapy did not improve survival (Figure 4E).

## 4 | DISCUSSION

Outcomes for older patients with PCNSL remain suboptimal, and our modern dataset confirms this finding with a median PFS of 17 months and median OS of 43 months, similar to data from both prior retrospective studies and clinical trials.^2,13^ We found that geriatric assessments, namely CIRS-G score, can be useful prognostic factors and that multi-agent chemotherapy regimens offer benefits compared to methotrexate alone. Additionally, post-induction therapy, in particular maintenance treatment, appeared to improve outcomes.

Significant uncertainty has existed in choosing the optimal induction regimen for older patients with PCNSL. While trials have definitively shown in a sequential, randomized fashion the benefits of adding cytarabine and then thiotepa to a HD-MTX backbone in younger patients,^6,7^ there is a paucity of such data in older patients. A French study led by ANOCEF-GOELAMS randomized patients to methotrexate/temozolomide (MT) vs an MPV-based regimen with cytarabine.^13^ Of note, rituximab was not included. That study favored MPV as the better regimen, with 2-year OS of 58% compared to 39% in the MT group, although these differences were not clearly statistically significant and the study was not powered for this purpose. Our data show better outcomes for both these regimens and longer survival in patients receiving MTR compared to MPV. Additionally, our data showed a benefit with combination regimens including HD-MTX as opposed to HD-MTX alone.

Our data also delineated a benefit regarding the inclusion of rituximab in induction therapy. The meta-analysis by Kasenda et al. of patients ages 60–90 found similar results, depending on statistical OS (H). interpretation.^14^ In contrast, the only randomized trial assessing the impact of rituximab found no benefit; however, this study included a younger patient population than our analysis and incorporated a more intensive chemotherapy backbone than is typically given to older patients (methotrexate, carmustine, teniposide prednisone). These factors may account for the lack of benefit noted with rituximab. In an unadjusted analysis, patients treated with combination regimens involving HD-MTX only had a tendency towards better OS, but this association was indeed statistically significant on adjusted multivariate analysis, and when the analysis was limited to patients treated after 1997 or treated in prospective trials. Few patients in our analysis received relatively intensive regimens such as methotrexate with high-dose cytarabine or MATRIX, in contrast to a retrospective study of 244 consecutive older patients in the United Kingdom, 20% of whom each received MATRIX and methotrexate/high-dose cytarabine,^15^ Outside of these studies, few other comparative data exist regarding optimal induction regimens for older patients.

There is also a paucity of data regarding the best consolidation or maintenance strategies in older PCNSL patients. A number of studies have investigated ASCT, mainly in younger patients. The IELSG 32 study enrolled 277 patients ages ≤70 with at least stable disease after induction and randomized them between ASCT with carmustine/thiotepa conditioning or whole brain radiation therapy (WBRT).^16^ PFS was similar between the two groups, but greater neurotoxicity was seen in the WBRT group, which would raise great concern in older patients. The Alliance group has recently presented CALGB 51101, which included patients up to age 75 who were randomized to MTR with cytarabine induction followed by either carmustine/thiotepa with ASCT or a cycle of non-myeloablative consolidation with cytarabine/etoposide.^17^ PFS from randomization was improved in the ASCT group, but only a non-significant trend towards improved PFS was seen with ASCT when calculated from the time of consolidation. While older patients are able to tolerate HD-MTX-based induction regimens, not all can tolerate these intensive consolidation strategies, and their applicability likely is limited to patients at the younger end of the age spectrum. The median age of patients in our analysis who underwent ASCT was 66 years. Patients in our analysis who underwent ASCT had an improved PFS compared to those who did not, although the OS difference was not statistically significant when adjusted for pre-treatment prognostic variable. Cytarabine consolidation appeared to improve OS but not PFS. Of note, the cytarabine doses and delivery in these older patients were less intensive than that used in treatment programs such as CALGB 50202, which prospectively studied a consolidation combination of cytarabine and etoposide in a younger patient population.^18^

No prospective comparative data exist regarding optimal maintenance strategies in older patients who cannot tolerate intensive consolidation.^3^ The NRG Oncology RTOG 0227 study was a phase I/II trial that assigned 53 patients in the phase II portion, 38% of whom were ≥age 60, to treatment with MTR followed by WBRT and then temozolomide 200 mg/m^2^ on days 1–5 every 4 weeks for 10 cycles.^19^ Two-year OS and PFS were approximately 80 and 60%, respectively. The Nordic Lymphoma Group treated 27 patients ages 66–75 with temozolomide maintenance dosed at 150 mg/m^2^ on days 1–5 every 28 days for 1 year or until disease progression.^20^ Two-year OS was 60%. The German PRIMAIN study treated 107 patients older than age 65 years with six monthly cycles of maintenance procarbazine (days 1–5) after initial induction with rituximab/methotrexate/procarbazine, resulting in a 2-year OS of 47%.^21^ Additionally, as lenalidomide has demonstrated activity in relapsed/refractory PCNSL with relatively good tolerability, a retrospective review of 13 patients older than age 70 treated at the University of California, San Francisco with maintenance lenalidomide dosed 5–10 mg daily showed that median PFS was not reached with median follow-up exceeding 30 months.^22^ Lenalidomide and temozolomide were the most common maintenance regimens administered to patients in our study. Maintenance therapy resulted in improved OS and PFS, highlighting the importance of this strategy to be further explored in future randomized trials in older patients. As our data suggest a more striking benefit for both ASCT and maintenance therapy in patients in PR after induction as opposed to CR, patients in PR should be especially considered for investigative approaches after induction.

Consolidative radiation therapy raises particular concerns regarding neurotoxicity in older patients. This approach did not show a survival benefit in our analysis, which adds to evidence that dampens enthusiasm about such a consolidation strategy. TRM in our data set was 6.9%. This figure is consistent with that seen in the UK retrospective analysis, of 6%^15^ and in prospective trials that have included younger patients.^7,8,23^

Prior studies have evaluated baseline clinical characteristics to develop models predicting long-term outcomes in PCNSL. However, these studies have included primarily younger and fit patients, who were able to undergo intensive induction regimens. The IELSG model, published in 2003,^24^ found that age greater than 60 years, ECOG PS more than 1, elevated LDH level, high CSF protein concentration, and deep brain involvement by lymphoma were significantly associated with worse survival. The Nottingham/Barcelona model incorporated age, PS, and the presence of multifocal lesions or leptomeningeal disease to develop a prognostic score.^25^ Memorial Sloan Kettering Cancer Center (MSKCC) developed a model incorporating age and PS.^26^ Ahn et al applied the variables in the Nottingham/Barcelona and MSKCC models to a single-center Korean cohort ages 29–77 and found that older age, multifocal lesions, and elevated CSF protein correlated with worse survival.^27^ Similarly, the Taipei Score is based on a multivariate analysis of age greater than or equal to 80 years, presence of deep brain lesions, and ECOG PS greater than or equal to 2.^28^ A large meta-analysis by Kasenda et al. also found advanced age and worse PS to be worse prognostic factors.^14^

Finally, the importance of geriatric assessments has been studied in lymphoma. Italian investigators have found that geriatric assessments done prior to therapy initiation correspond with the receipt of aggressive versus less aggressive therapy, as well as long-term survival.^29,30^ The Fondazione Italiana Linfomi (FIL) developed the initial geriatric assessment tool in older patients with lymphoma, incorporating scores from Cumulative Illness Rating Scale-Geriatric (CIRS-G), ADL, Instrumental Acitivities of Daily Living (IADL) and age, classifying patients into categories of fit, unfit, and frail, and thereby providing a more robust and nuanced assessment than PS alone.^31,32^ Our data support the importance of geriatric assessments in older patients with PCNSL. This should be further examined in prospective studies.

Limitations of our study include the retrospective nature of the data and selection bias that is inherently present in such studies, particularly when analyzing data about consolidation and maintenance approaches, as we only analyzed these strategies in patients who achieved a response to initial therapy. It is unlikely that treating physicians of patients in our data set prospectively gathered information on geriatric assessments at the time of deciding induction treatment, and so decisions about induction therapy likely were made based on subjective estimates of patient frailty and ability to tolerate chemotherapy. We attempted to account for such subjectivity by adjusting survival data for pre-treatment prognostic factors.

Altogether, our data indicate that outcomes in older PCNSL patients appeared optimized with the use of HD-MTX combination induction regimens as well as maintenance therapy. Furthermore, our retrospective analysis suggests an apparent benefit for patients who received maintenance therapies. Future clinical trials should focus on these findings. In addition, we identified several prognostic factors, including geriatric measures, associated with divergent patient outcomes. Future prospective studies for older patients with PCNSL are needed to optimally tailor therapy leveraging geriatric assessments.

## Figures and Tables

**TABLE 1:** Baseline patient and disease characteristics (continuous variables).

Variable	Data available (%)	Median	PFS			OS		
	
			HR	95% CI	*p*	HR	95% CI	*p*
Age (yr)	100	70.7	1.042	1.026–1.058	<.001	1.049	1.032–1.068	<.001
Creatinine clearance (ml/min)	60	81	1.007	1.002–1.01	.003	1.008	1.003–1.01	.0014
Hemoglobin (g/dl)	95	12.9	1.103	1.04–1.17	.001	1.14	1.06–1.21	<.001
Albumin (g/dl)	92	3.5	1.579	1.302–1.91	<.001	1.916	1.553–2.370	<.001
Ki-67 (%)	53	80	1.004	0.997–1.012	.2863	1.004	0.995–1.012	0.3678
CIRS-G	91	6	1.030	1.009–1.052	.0050	1.042	1.019–1.066	.0004

**TABLE 2:** Baseline patient and disease characteristics (categorical variables).

			PFS			OS		
		
Categorical variable	*N*	%	HR	95% CI	*p*	HR	95% CI	*p*
**LDH elevated**								
Yes	157	29	1.202	0.948–1.523	0.13	1.148	0.875–1.507	0.026
No	287	53						
NA	95	18						
**Prior or concurrent malignancy**								
Yes	122	23	1.074	0.839–1.375	0.572	1.148	0.875–1.507	.3188
No	414	77						
NA	3	.006						
**Prior organ transplant**								
Yes	43	8	0.933	0.626–1.391	0.732	1.149	0.756–1.745	.516
No	494	92						
NA	2	.03						
**CSF protein elevated**								
Yes	233	43	1.157	0.872–1.536	.311	0.953	0.697–1.303	0.764
No	111	20						
NA	195	36						
**CSF involved**								
Yes	68	13	0.881	0.637–1.219	.444	1.043	0.736–1.477	.815
No	325	60						
NA	146	27						
**Ocular involvement**								
Yes	52	10	0.938	0.659–1.337	.724	0.780	0.515–1.182	.242
No	385	71						
NA	102	19						
**Cell of origin**								
GC	70	13						
Non-GC	219	40	0.858	0.620–1.187	.355	0.880	0.610–1.270	.495
NA	250	46						
**Double expressor (cmyc and bcl2) by IHC**								
Yes	74	14	0.8	0.542–1.181	.2618	0.890	0.573–1.382	.604
No	108	20						
NA	357	66						
***MYC* translocation by FISH**								
Yes	27	5	1.791	1.11–2,89	.0169	1.634	0.917–2.909	.0954
No	128	24						
NA	384	71						
**EBER**								
Positive	33	6	.764	.476–1.228	.2666	.890	.538–1473	.6501
Negative	279	52						
NA	227	42						
**Number of parenchymal sites**								
1	239	44	1.014	.820–1.255	.8983	.935	.736–1.188	.5816
>1	271	50						
NA	29	5						
**Geriatric syndrome**								
Yes	247	46	1.402	1.137–1.729	.0016	1.625	1.283–2.058	<.0001
No	282	52						
NA	10	2						
**Impairment in any ADL**								
Yes	193	36	1.403	1.127–1.745	.0024	1.712	1.341–2.187	<.0001
No	314	58						
NA	32	6						
**ECOG PS**								
0	60	11						
1	162	31	1.062	.728–1.548	.7555	1.453	.896–2.357	.130
2	154	29	1.378	.947–2.005	.0942	2.051	1.274–3.300	.0031
3	66	12	1.576	1.026–2.422	.0377	2.817	1.677–4.733	<.0001
4	25	5	3.238	1.873–5.596	<.0001	5.655	3.052–10.48	<.0001
NA	72	13						

**TABLE 3:** Multivariate analysis of prognostic factors (all patients, at diagnosis).

	PFS			OS		
		
Variable	HR^a^	95% CI	*p* value	HR^a^	95% CI	*p* value
**Age** Increased yearly >age 60 years	1.044	1.018–1.070	.0008	1.047	1.019–1.075	.0008
**ECOG PS** 0/1 versus 2–4	0.723	0.577–0.906	.0049	0.552	0.412–0.740	<.0001
**Hypoalbuminemia** Yes versus no	NA			1.359	1.009–1.831	.0438
**CIRS-G Score**	NA			1.037	1.005–1.070	.0241

a Hazard ratios >1 indicate a factor with poor prognosis, whereas those <1 indicate a factor with favorable prognosis.
